# Budd-Chiari primitif: défi diagnostique et thérapeutique

**DOI:** 10.11604/pamj.2020.37.142.25109

**Published:** 2020-10-08

**Authors:** Fatimaezzahra Benali, Nourdin Aqodad

**Affiliations:** 1 Centre Hospitalier Régional de Geulmim, Geulmim, Maroc,; 2 Faculté de Médecine et de Pharmacie, Université Ibn Zohr Agadir, Service de Gastroentérologie, Centre Hospitalier Régional Hassan II, Agadir, Maroc

**Keywords:** Budd-Chiari primitif, étiologies, traitement, Primitive Budd-Chiari syndrome, etiologies, treatment

## Abstract

Le syndrome de Budd-Chiari primitif est une hépatopathie caractérisée par une obstruction du flux veineux hépatique dans l´espace vasculaire situé entre les veinules hépatiques et la jonction entre la veine cave inférieure et l´oreillette droite excluant donc les causes d´amont (syndrome d´obstruction sinusoïdale) et d´aval (causes cardiaques). Cette obstruction veineuse endoluminale est due principalement à une thrombose ou à sa séquelle fibreuse. C´est une affection rare, qui prédomine chez l´adulte jeune. Les manifestations cliniques sont extrêmement variables, elle peut être asymptomatique, aiguë, subaiguë ou chronique. Le diagnostic repose essentiellement sur l´échographie doppler et/ou l´imagerie par résonance magnétique. Plusieurs étiologies ont été identifiées, en particulier, le syndrome myéloproliferatif, le syndrome des antiphospholipides, l´hémoglobinurie paroxystique nocturne, les affections thrombogènes héréditaires... Le traitement est étiologique et symptomatique, les recommandations thérapeutiques sont organisées en algorithme. Le pronostic est nettement amélioré grâce aux avancées thérapeutiques récentes.

## Abstract (en)

Primary Budd-Chiari syndrome is an hepatopathy characterized by obstruction of the hepatic venous flow in the vascular space between the hepatic venules and the junction between the inferior vena cava and the right atrium, thus excluding the causes of upstream (sinusoidal obstruction syndrome) and downstream (cardiac causes). This endoluminal venous obstruction is mainly due to thrombosis or its fibrotic consequences. This is a rare disease, mainly affecting young adults. Clinical manifestations are extremely variable; it can be asymptomatic, acute, subacute or chronic. The diagnosis is mainly based on Doppler ultrasound and/or magnetic resonance imaging. Several causes have been identified, in particular, myeloproliferative syndrome, antiphospholipid syndrome, paroxysmal nocturnal hemoglobinuria, inherited thrombotic disorders. Symptomatic and etiologic treatments are the commonly used approaches, treatment recommendations are organized in algorithm. Recent therapeutic advances can significantly improve the prognosis.

## Introduction

Le syndrome de Budd-Chiari est une hépatopathie caractérisée par une obstruction du flux veineux hépatique dans l´espace vasculaire situé entre les veinules hépatiques et la jonction entre la veine cave inférieure et l´oreillette droite, excluant donc les causes d´amont (syndrome d´obstruction sinusoïdale) et d´aval (causes cardiaques). Le syndrome de Budd-Chiari primitif est dû à l´obstruction veineuse endoluminale par thrombose ou à sa séquelle fibreuse, révélant un état pro-thrombotique sous-jacent. Le syndrome de Budd-Chiari (SBC) secondaire est défini par une obstruction par du matériel d'origine extravasculaire. Cette entité sera exclue du champ de notre discussion. Plusieurs avancées diagnostiques et thérapeutiques ont été réalisées dernièrement. Le but de cet article est de mettre le point sur ces actualités d´ordre pratique.

## Seminar

**Historique et épidémiologie:** le syndrome de Budd-Chiari est nommé en hommage au physicien George Budd et au pathologiste Hans Chiari. En 1845, George Budd a défini le syndrome comme étant une maladie du foie associant hépatomégalie, ascite et douleur abdominale; et ce n´est qu´en 1899, que Hans Chiari a réussi à le décrire sur le plan histopathologique, en se basant sur des études nécropsiques. Il s´agit d´une maladie rare dont la prévalence et l´incidence varient dans le monde, elle est de 0,1 à 10 par million d´habitants par an [1, 2] plus fréquente en Asie [3].

**Histopathologie des lésions des veines hépatiques et de la veine cave inférieure:** la thrombose pure est rare. Dans la majorité des cas, la lésion élémentaire est la thrombose veineuse, qui évolue vers une fibrose séquellaire. Avec le temps, le thrombus se réorganise en tissu fibreux entraînant soit un rétrécissement localisé de la veine thrombosée, soit une oblitération diffuse la transformant en un cordon fibreux. Les sténoses localisées peuvent prendre l'aspect d'une membrane.

**Physiopathologie et manifestation clinique:** l´obstruction veineuse hépatique a trois conséquences dont découlent les manifestations cliniques: une diminution de la perfusion hépatique, inconstante et habituellement transitoire par le développement de la circulation collatérale, l´hypertrophie compensatrice du segment I, l´augmentation de pression portale et l´augmentation du débit de l´artère hépatique, cette hyper-artérialisation des zones déportalisées entraine l´apparition des nodules HNF-like. Une augmentation de la pression sinusoïdale entraine en premier lieu une dilatation sinusoïdale et une congestion et par conséquent une augmentation du volume du foie et de la perméabilité des parois sinusoïdales au liquide interstitiel d´où découlent l´hépatomégalie et l´ascite. En second lieu, une hypertension portale se développe progressivement et par conséquent une circulation veineuse collatérale. Un syndrome phlébitique qui associe fièvre, douleurs et syndrome inflammatoire.

**Diagnostic:** un syndrome de Budd-Chiari primitif doit être toujours suspecté devant: une hépatomégalie, ascite et douleurs abdominales hautes; une insuffisance hépatique fulminante avec hépatomégalie et ascite; une maladie chronique du foie d´étiologie inexpliquée et une maladie du foie chez un patient connu porteur d´une affection thrombogène.

***Le bilan biologique:*** les perturbations du bilan biologique sont essentiellement liées à la vitesse et à l´étendue de l´obstruction veineuse. Dans les formes aigues l´insuffisance hépatocellulaire peut être sévère, rarement fulminante ou su fulminante avec des transaminases élevées, supérieures à 5 fois la normale, un taux de prothrombine ≤50%, une ascite riche en protides et une insuffisance rénale. Dans les formes chroniques la cytolyse est souvent inférieure à 5 fois la normale, le taux de prothrombine est ≥50%, une hypoalbuminémie et une insuffisance rénale sont fréquentes.

**Figure 1:**
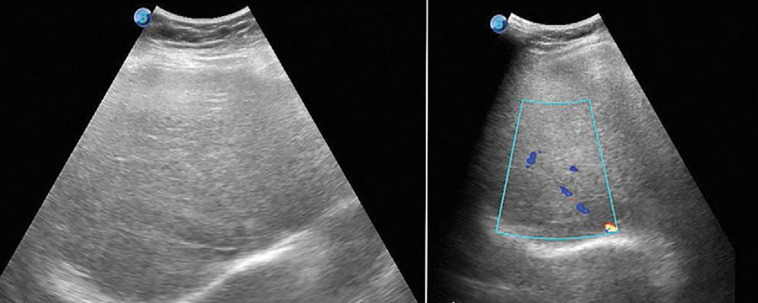
échographie abdominale montrant une sténose de la veine sus-hépatique droite

***L´échographie-doppler:*** l´obstruction des principales veines hépatiques peut être reconnue par la seule échographie-doppler dans plus de 80% des cas. Les thromboses anciennes se présentent sous forme d´un fin cordon hyperéchogène sur le trajet de la veine correspondante. Les thromboses récentes se présentent sous forme d'une veine élargie remplie de matériel hypoéchogène. L'échographie-doppler pulsé et couleur montre un flux démodulé de vitesse continue dans la veine en amont de la sténose au lieu de la morphologie triphasique habituelle. En doppler couleur, on visualise les voies de dérivation du retour veineux (Figure 1). Les formes avec atteinte de la terminaison de la veine cave peuvent se manifester par un flux inversé dans la veine cave et des lésions variables des veines hépatiques [4, 5].

***La tomodensitométrie (TDM):*** la TDM a peu d'intérêt dans cette pathologie. Les lésions sténos antes des veines sus-hépatiques sont mal visibles, même si les thromboses peuvent être mises en évidence. Seuls les signes indirects comme la dysmorphie, l'ascite et un parenchyme hépatique hétérogène après injection de produit de contraste orientent vers ce diagnostic. Elle trouve sa place dans le syndrome de Budd-Chiari secondaires aux tumeurs hépatiques ou rétro-péritonéales.

***L´imagerie par résonance magnétique (IRM):*** l´IRM ou le scanner sont indiqués dans les formes sévères, ou peut se discuter une greffe hépatique en urgence et dans les formes chroniques ou le tableau clinique est ambigu. Une double sémiologie est retrouvée, celle du parenchyme hépatique et celle des vaisseaux.

*Anomalies parenchymateuses:* un hypersignal sur les séquences pondérées en T2 est retrouvé sur les segments atteints à la phase aiguë, il est secondaire à la congestion de la phase aiguë. Dans les phases chroniques, au contraire, le signal du foie baisse par rapport à celui de la rate ou du segment I.

*Anomalies vasculaires:* on retrouve la sémiologie de la thrombose vasculaire; signal élevé sur les séquences en écho de spin et absence de signal en écho de gradient [5].

***La biopsie hépatique:*** les techniques d´imageries non invasives restent moins sensibles pour le diagnostic des lésions des veines hépatique de petit calibre, et par conséquent une biopsie hépatique s´avère indispensable.

### Formes cliniques

Plusieurs formes cliniques peuvent être individualisées, et elles sont vraisemblablement liées à la vitesse et à l´étendue de l´obstruction veineuse.

***Forme asymptomatique:*** cette forme représente environ 20% des cas du syndrome du Budd-Chiari [3]. Elle est caractérisée par l´absence de signes cliniques, expliquée probablement par la perméabilité d´une des grosses veines hépatiques ou le développement de très grosses veines collatérales.

***SBC aigu:*** cette entité rare correspond éventuellement à une obstruction simultanée des 3 veines sus-hépatiques en l´absence de maladie hépatique sous-jacente. Elle est caractérisée par la constitution en quelques jours d´une insuffisance hépatique qui peut être parfois sévère. Il existe une hépatomégalie et des transaminases élevées supérieures à 5 fois la normale. Il n´y a pas de dysmorphie hépatique. Une ascite et une insuffisance rénale sont très fréquentes.

***Forme chronique:*** elle représente environ 60% des cas de syndrome de Budd-Chiari. Cette entité est caractérisée par une dysmorphie hépatique, un réseau veineux collatéral typique, des transaminases inferieures à 5 fois la normale et l´apparition progressive d´une ascite.

***Forme subaiguë:*** cette entité est caractérisée par au moins 1 des éléments de SBC aigu associé à au moins 1 des éléments de SBC chronique, Il existe une dysmorphie hépatique. Les manifestations datent généralement de moins de 2 mois quand le diagnostic est fait.

***Atteinte de la veine cave inférieure:*** une obstruction membraneuse de la veine cave inférieure peut être isolée ou associée à une obstruction des veines sus-hépatiques [6].

**Etiologies du syndrome de Budd-Chiari primitif:** la thrombose est associée dans 2/3 des cas à une ou plusieurs affections prothrombotiques sous-jacentes (Tableau 1). Dont le mécanisme étiopathologique demeure à ce jour ambigu [2, 3].

**Tableau 1:** bilan étiologique du syndrome de Budd-Chiari primitif

	Interrogatories
A-Bilan de première ligne	-Antécédents personnels ou familiaux de thromboses veineuses profondes
	-Notion de prise de contraception orale
	**Bilan biologique**
	-Recherche de mutation JAK 2 dans le sang périphérique. Si elle est indétectable, une biopsie ostéo-médullaire est indispensable (syndromes myéloproliferatifs)
	-Recherche du déficit en CD55 and CD59 par cytométrie en flux (hémoglobinurie paroxystique nocturne)
	- Résistance à la protéine C activée. Recherche de la mutation du facteur V de Leiden
	-Recherche de la mutation du gène de la prothrombine
	-Anticoagulant lupique, anticorps anti beta2 glycoprotein-1, anticorps anticardiolipine (syndrome des antiphospholpides)
B-Bilan de deuxième ligne	-Dosage sérique de la protéine C, protéine S et de l´antithrombine
	-Dosage sérique de l´homocystéine

### Les étiologies thrombogènes acquises

***Les syndromes myéloprolifératifs (SMP):*** ce sont des maladies chroniques caractérisées par une prolifération clonale des cellules myéloïdes précurseurs soit des lignées granulocytaires: leucémie myéloïde chronique, soit des lignées érythroïdes: polyglobulie de Vaquez ou bien des lignées mégacaryocytaires: thrombocytémie essentielle. Ils sont la cause la plus fréquemment retrouvée du syndrome de Budd-Chiari primitif. De ce fait, ils doivent être systématiquement recherchés.

***Le syndrome des antiphospholipides (SAPL):*** le syndrome des antiphospholipides est caractérisé par l'association d'évènements cliniques (thromboses et/ou complications obstétricales) et de la présence d'autoanticorps hétérogènes. Ces derniers entraînent l'inhibition des acteurs naturels contrôlant le risque de coagulation excessive. Ce mécanisme est probablement à l'origine de l'état prothrombotique des patients.

***L´hémoglobinurie paroxystique nocturne:*** cette affection est causée par un défaut de protection de la membrane des hématies contre la lyse par le complexe d'attaque membranaire du complément. La triade clinique classique associe une hémolyse intravasculaire, une hypoplasie médullaire de degré variable et des accidents thromboemboliques, de localisations souvent atypiques: les veines du système porte, le syndrome de Budd-Chiari et les localisations cérébrales.

***Les étiologies thrombogènes héréditaires:*** les anomalies les plus fréquemment associées à la maladie thromboembolique veineuse sont la mutation Leiden du facteur V, la mutation du facteur II G20210A, ainsi que les déficits en antithrombine, protéine C et protéine S. Toutefois, leur prévalence reste modeste par rapport aux anomalies acquises. Des anomalies du facteur II et du métabolisme de l´homocystéine peuvent également être relevées. A noter que ces inhibiteurs de la coagulation sont synthétisés par le foie, et par conséquent, l´atteinte hépatique due au syndrome de Budd-Chiari entraîne une baisse non spécifique des taux plasmatiques.

***Médicaments:*** le Dacarbazine peut induire une forme fulminante d´occlusion des veines hépatiques. L´utilisation des contraceptifs oraux augmente le risque d´occlusion des veines hépatiques.

***Grossesse et post-partum:*** plusieurs cas d´occlusions des veines hépatiques survenant pendant la grossesse ou les suites de couches, ont été rapportés. Une affection thrombogène sous-jacente doit donc être toujours suspectée.

***Autres affections thrombogènes systémiques:*** maladie de Behçet, la colite ulcéreuse, la sarcoïdose, maladie cœliaque.

***Syndrome de Budd-Chiari de cause inconnue:*** la proportion des cas idiopathiques descend à moins de 10%.

**Traitement:** en Europe, les recommandations thérapeutiques sont organisées en algorithme, d´abord un traitement médical à base d´anticoagulants est instauré chez tous les patients, si échec une re-perméabilisation endovasculaire par angioplastie, stent ou thrombolyse est réalisée. En cas de persistance ou d´aggravation des symptômes, une dérivation porto-systémique par TIPS est alors envisagée, et enfin la transplantation hépatique est proposée comme dernière alternative thérapeutique. Cependant, en Asie, la majorité des patients sont traités d´emblée par une recanalisation percutanée ou bien un TIPS, le traitement médical est réservé aux patients exclus du traitement percutané [7].

**Traitement médical:** le traitement de la maladie causale doit être démarré sans délai. Un traitement anticoagulant à base d´héparine de bas poids moléculaire à dose curative puis relai par les anti-vitamines K (AVK) doit être instauré chez tous les patients et sans délai, dans le dessein de prévenir l´extension de la thrombose, et la survenue d´un autre évènement thromboembolique. L´INR doit être entretenue entre 2 et 3. La durée du traitement est précisée en fonction de l´étiologie et peut être prolongée à vie. Peu de données concernant l´utilisation du rivaroxaban chez les patients avec un Budd-Chiari primitif.

**La re-perméabilisation des veines hépatiques et de la VCI:** les patients qui ne répondent pas au traitement médical, sont candidats à une thrombolyse pharmacologique, à une angioplastie transluminale percutanée ou aux deux à la fois. Le choix entre ces techniques doit être discuté dans un centre spécialisé. La thrombolyse pharmacologique isolée par voie générale ne donne pas de résultats satisfaisants. L´angioplastie peut être précédée d´une thrombolyse in situ quand la veine atteinte est thromboses [2, 6].

**Le shunt porto systémique intrahépatique ou le TIPS:** en effet, le TIPS peut être réalisé d´emblée chez les patients ayant développés une cirrhose ou bien après échec des techniques de re-perméabilisation percutanées. Une étude européenne rétrospective multicentrique, incluant 124 patients traités par TIPS, a montré une excellente survie sans transplantation à 1 et 5 ans (88% et 78%, respectivement) [8].

**La transplantation hépatique:** la transplantation hépatique est indiquée chez les patients ayant une cirrhose sévère, une insuffisance hépatique fulminante, ou bien en échec de TIPS. La survie à 5 ans après transplantation hépatique pour un syndrome de Budd-Chiari est de 90% [8].

**Pronostic:** en dehors du MELD et du Child-Pugh, plusieurs scores pronostics ont été établis, entre autres, le score de Rotterdam, score de Clichy, ou le score BCS-TIPS (Budd-Chiari Syndrome-Transjugular Intrahépatique Portosystemic Shunt). Toutefois, la prédiction individuelle du pronostic reste difficile, vu l´hétérogénéité du syndrome de Budd-Chiari, essentiellement clinique et étiologique.

## Conclusion

Le pronostic du syndrome de Budd-Chiari primitif s´est nettement amélioré au cours des dernières années. L´histoire naturelle demeure inconnue. Les étiologies sont multiples dominées par les syndromes myéloproliferatifs. Le diagnostic repose essentiellement sur l´échographie-doppler des veines sus hépatiques et de la veine cave inférieur. La prise en charge thérapeutique doit être précoce et multidisciplinaire.
